# Supplementary material for: Monoallelically expressed noncoding RNAs form nucleolar territories on NOR-containing chromosomes and regulate rRNA expression
Source: eLife. 2024 Jan 19;13:e80684. doi: 10.7554/eLife.80684 (PMC10852677; doi:10.7554/eLife.80684)
Supplement: Figure 5—source data 2. [file elife-80684-fig5-data2.zip › Figure 5-Source Data 2/Figure 5J_Northern bolt with labels.docx]

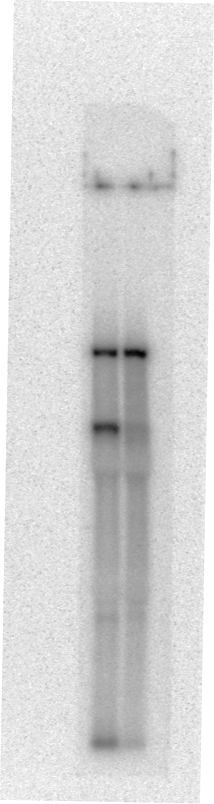


Northern blot using 5-ETS-1 probe from total RNA isolated from control and SNUL-depleted WI-38 cells

47S

30S^+1^

+1-01

ASO-SNUL

Ctr-ASO
