# Supplementary figures and images for: Monoallelically expressed noncoding RNAs form nucleolar territories on NOR-containing chromosomes and regulate rRNA expression
Source: eLife. 2024 Jan 19;13:e80684. doi: 10.7554/eLife.80684 (PMC10852677; doi:10.7554/eLife.80684)

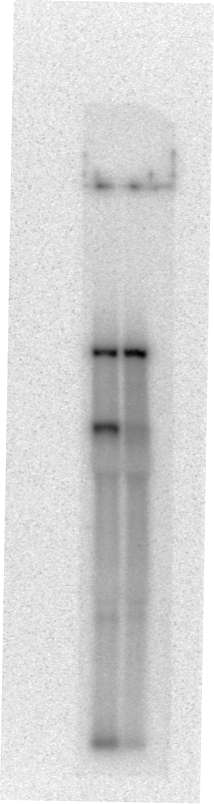

Supplement: Figure 5—source data 2. [file elife-80684-fig5-data2.zip › Figure 5-Source Data 2/Figure 5J_Northern bolt original file.tif]

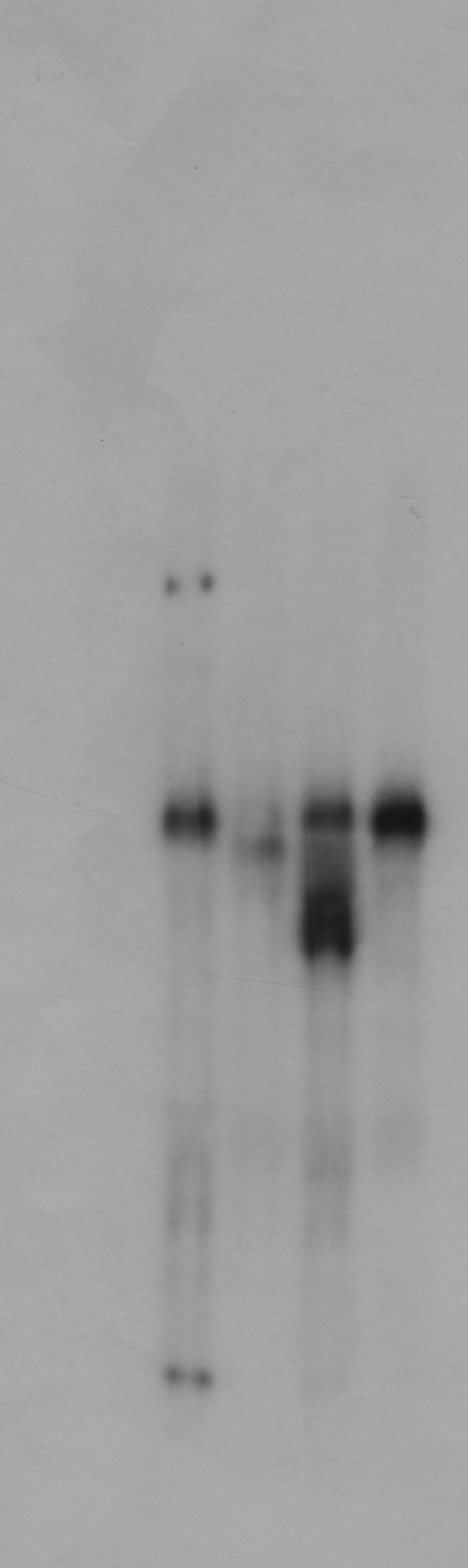

Supplement: Figure 5—figure supplement 2—source data 1. [file elife-80684-fig5-figsupp2-data1.zip › Figure 5-figure supplement 2-Source Data 1/Figure 5-figure supplement2C_Northern bolt original file.tiff]
