# Supplementary material for: Monoallelically expressed noncoding RNAs form nucleolar territories on NOR-containing chromosomes and regulate rRNA expression
Source: eLife. 2024 Jan 19;13:e80684. doi: 10.7554/eLife.80684 (PMC10852677; doi:10.7554/eLife.80684)
Supplement: Figure 5—figure supplement 2—source data 1. [file elife-80684-fig5-figsupp2-data1.zip › Figure 5-figure supplement 2-Source Data 1/Figure 5-figure supplement2C_Northern bolt with labels.docx]

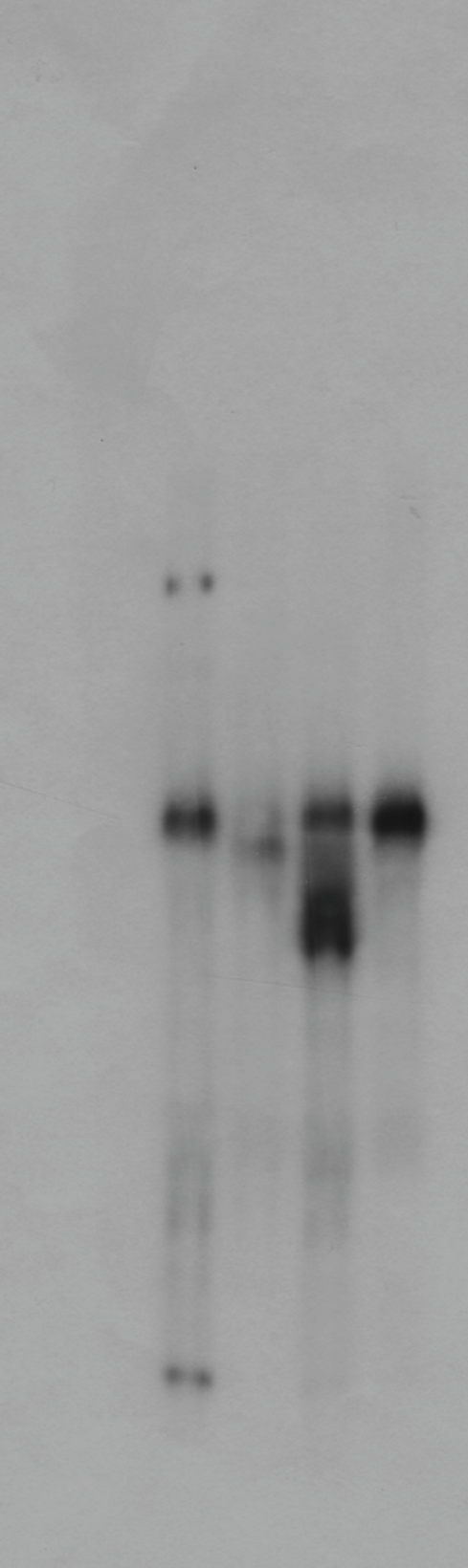


Northern blot using 5-ETS-1 probe and total RNA isolated from control SNUL-depleted, 18S knock down and ITS-1 knock down WI-38 cells

ASO-ITS1

ASO-18S

ASO-SNUL

Ctr-ASO

+1-01

30S^+1^

47S
